# Central tolerance is impaired in the middle‐aged thymic environment

**DOI:** 10.1111/acel.13624

**Published:** 2022-05-13

**Authors:** Jessica N. Lancaster, Damaris E. Keatinge‐Clay, Jayashree Srinivasan, Yu Li, Hilary J. Selden, Seohee Nam, Ellen R. Richie, Lauren I. R. Ehrlich

**Affiliations:** ^1^ 12330 Department of Molecular Biosciences The University of Texas at Austin Austin Texas USA; ^2^ 4002 Department of Epigenetics and Molecular Carcinogenesis The University of Texas MD Anderson Cancer Center Houston Texas USA; ^3^ Department of Oncology Dell Medical School at The University of Texas at Austin Austin Texas USA; ^4^ Present address: Department of Immunology The Mayo Clinic Scottsdale Arizona USA

**Keywords:** cellular immunology, central tolerance, immune aging, T cell, thymus involution

## Abstract

One of the earliest hallmarks of immune aging is thymus involution, which not only reduces the number of newly generated and exported T cells, but also alters the composition and organization of the thymus microenvironment. Thymic T‐cell export continues into adulthood, yet the impact of thymus involution on the quality of newly generated T‐cell clones is not well established. Notably, the number and proportion of medullary thymic epithelial cells (mTECs) and expression of tissue‐restricted antigens (TRAs) decline with age, suggesting the involuting thymus may not promote efficient central tolerance. Here, we demonstrate that the middle‐aged thymic environment does not support rapid motility of medullary thymocytes, potentially diminishing their ability to scan antigen presenting cells (APCs) that display the diverse self‐antigens that induce central tolerance. Consistent with this possibility, thymic slice assays reveal that the middle‐aged thymic environment does not support efficient negative selection or regulatory T‐cell (Treg) induction of thymocytes responsive to either TRAs or ubiquitous self‐antigens. This decline in central tolerance is not universal, but instead impacts lower‐avidity self‐antigens that are either less abundant or bind to TCRs with moderate affinities. Additionally, the decline in thymic tolerance by middle age is accompanied by both a reduction in mTECs and hematopoietic APC subsets that cooperate to drive central tolerance. Thus, age‐associated changes in the thymic environment result in impaired central tolerance against moderate‐avidity self‐antigens, potentially resulting in export of increasingly autoreactive naive T cells, with a deficit of Treg counterparts by middle age.

## Graphical abstract

Age‐associated thymus involution impairs the capacity of the middle‐aged thymus to support central tolerance to self‐antigens with moderate TCR avidities, although tolerance to high avidity self‐antigens remains intact. The decline in both negative selection and generation of regulatory T cells by middle‐age is associated with reduced numbers and proportions of AIRE^+^ mTECs and MHCII^hi^ cDC1s.

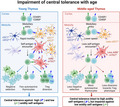

## 1 INTRODUCTION

Thymus involution begins in childhood, resulting in a progressive age‐associated reduction in the generation and export of naive T cells (Chinn et al., 2012). Diminished thymic output contributes to declining T‐cell immunity, a major driver of immune dysfunction in aged mice and humans (Elyahu & Monsonego, 2021; Nikolich‐Žugich, 2014). Nonetheless, the thymus continues to produce and export new T cells, albeit at reduced numbers, into advanced age (Flores et al., 1999; Hale et al., 2006; Lynch et al., 2009). Quantification of human T‐cell receptor excision circles (TRECs) indicates thymic output is detectable until ~80 years of age (Mitchell et al., 2010; Nasi et al., 2006). Although human naïve T cells are maintained largely by homeostatic proliferation (Mold et al., 2019), thymic output is required to sustain normal numbers of naive T cells in both mice and humans (Appay & Sauce, 2014; Bourgeois et al., 2008). The thymus remains the sole source of new T‐cell and Treg clones throughout life, yet little is known about the impact of thymus aging on qualitative changes in T‐cell maturation and selection.

As the thymus involutes and fewer T cells are exported, the peripheral T compartment is progressively comprised of phenotypic memory cells (Goronzy & Weyand, 2019; Nikolich‐Žugich, 2008; Srinivasan et al., 2021). This reduction in naïve T cells in the elderly contributes to increased susceptibility to infectious disease and decreased vaccine responsiveness (Nikolich‐Žugich, 2014). Interestingly, in middle‐age, although T‐cell export from the thymus declines substantially (Mold et al., 2019; den Braber et al., 2012; Ito et al., 2017), the incidence of new‐onset autoimmunity peaks (Watad et al., 2017). Also, both naive CD4^+^ and CD8^+^ T cells become more self‐reactive with age in mice (Deshpande et al., 2015; Quinn et al., 2016; Rudd et al., 2011), suggesting thymic selection could become impaired with age.

The thymic medulla is a specialized microenvironment for inducing T‐cell central tolerance. Following T‐lineage commitment, differentiation, and positive selection in the thymic cortex, developing T cells express chemokine receptors that promote their migration into the medulla (Cowan et al., 2014; Ehrlich et al., 2009; Hu et al., 2015; Kadakia et al., 2019; Kurobe et al., 2006; Lancaster et al., 2018), where they encounter numerous self‐antigens presented by mTECs and hematopoietic APCs (HAPCs), including conventional dendritic cells (cDCs), B cells, and plasmacytoid dendritic cells (pDCs). If the T‐cell receptors (TCRs) on a given thymocyte bind self‐peptide:MHC complexes on medullary APCs with sufficient strength, the thymocyte undergoes negative selection or diversion to the Treg lineage, enforcing central tolerance (Klein et al., 2014). Mature mTECs play an essential role in tolerance induction, as they collectively express ~90% of the proteome, including *Aire*‐dependent tissue‐restricted antigens (TRAs), which are otherwise expressed in only a few peripheral tissues (Bautista et al., 2021; Bornstein et al., 2018; Brennecke et al., 2015; Meredith et al., 2015; Sansom et al., 2014). Thymocytes must be tolerized to the full repertoire of mTEC‐derived self‐antigens to avoid autoimmunity (Aaltonen et al., 1997; Anderson, 2002; DeVoss et al., 2006; Nagamine et al., 1997), but a given TRA is expressed by only ~1%–3% of mTECs (Derbinski et al., 2005, 2008), creating a sparse mosaic of self‐antigen display in the medulla. Thymic cDCs also play a critical role in thymic tolerance by presenting self‐antigens acquired from circulation, peripheral tissues, and mTECs (Atibalentja et al. 2011; Bonasio et al. 2006; Perry et al., 2014, 2018; Leventhal et al., 2016; Watanabe et al. 2020; Vollmann et al. 2021). Thus, it is critical that post‐positive selection thymocytes efficiently enter the medulla and rapidly scan mTECs and HAPCs to encounter the complete arrays of self‐antigens that induce broad central tolerance.

Age‐associated changes in thymic APCs or expression of self‐antigens could impair central tolerance. Aging is associated with substantial changes in the thymic stromal compartment (Baran‐Gale et al., 2020; Chinn et al., 2012; Lynch et al., 2009; Srinivasan et al., 2021; Venables et al., 2019): the cortex thins (Venables et al., 2019), TEC proliferation and cellularity are reduced (Gray et al., 2006), and mTECs decline (Baran‐Gale et al., 2020; Chinn et al., 2012; Lepletier et al., 2019). Notably, expression of TRAs in the medulla diminishes with age (Griffith et al., 2012), and thymic B cells and DCs change in composition and molecular properties (Cepeda et al., 2018; van Dommelen et al., 2010; Flores et al., 2001; Ki et al., 2014; Nuñez et al., 2016; Varas et al., 2003).

The efficiency with which autoreactive thymocytes are negatively selected is dependent on the TCR‐binding avidity of the selecting self‐antigen. High‐avidity self‐antigens induce negative selection (Klein et al., 2019), while those of moderate avidity can allow reactive thymocytes to escape negative selection and cause autoimmunity (Koehli et al., 2014; Zehn & Bevan, 2006). Beyond TCR‐binding affinity, the pattern of self‐antigen expression in the thymus also modulates autoreactive thymocyte fates. Ubiquitously‐expressed self‐antigens induce more robust negative selection, while rare, *Aire*‐dependent TRAs induce both negative selection and Treg induction (Hassler et al., 2019; Malhotra et al., 2016). Given that aging results in diminished expression of TRAs and changes in the composition and organization of mTECs and HAPCs, autoreactive thymocytes in the aged microenvironment may be screened less efficiently against ubiquitous and/or rare, tissue‐specific self‐antigens. Little is known about the impact of aging on thymocyte selection. In a mouse model of accelerated involution, negative selection was impaired, but Treg induction was enhanced (Coder et al., 2015; Oh et al., 2017). In contrast, following natural aging, Treg generation was diminished (Thiault et al., 2015), which was attributed to increased re‐entry of peripheral Tregs into the thymus, which outcompeted resident Treg progenitors for IL‐2 (Hemmers et al., 2019; Weist et al., 2015). These findings raise questions about whether the aged thymus supports efficient negative selection and Treg induction against different types of self‐antigens.

In this study, we use live thymic slices in combination with 2‐photon microscopy (2PM) to test the ability of the naturally aged thymic environment to support thymocyte medullary entry and rapid motility, as well as negative selection and Treg induction to ubiquitous self‐antigens or model TRAs. We find that thymocytes, regardless of age, do not migrate as rapidly in a middle‐aged 12‐month‐old (MO) relative to a 1MO thymic environment. Furthermore, the middle‐aged thymus does not support efficient negative selection or Treg induction of thymocytes responsive to self‐antigens of moderate avidities. However, central tolerance, including Treg induction, remains intact for thymocytes responsive to ubiquitous high‐affinity self‐antigens in the middle‐aged thymus. Thus, the middle‐aged thymus does not support efficient central tolerance to moderate‐avidity self‐antigens, possibly resulting in export of poorly tolerized T cells to the periphery by middle‐age.

## 2 RESULTS

### 2.1 The middle‐aged thymus does not support rapid motility of medullary thymocytes

**FIGURE 1:**
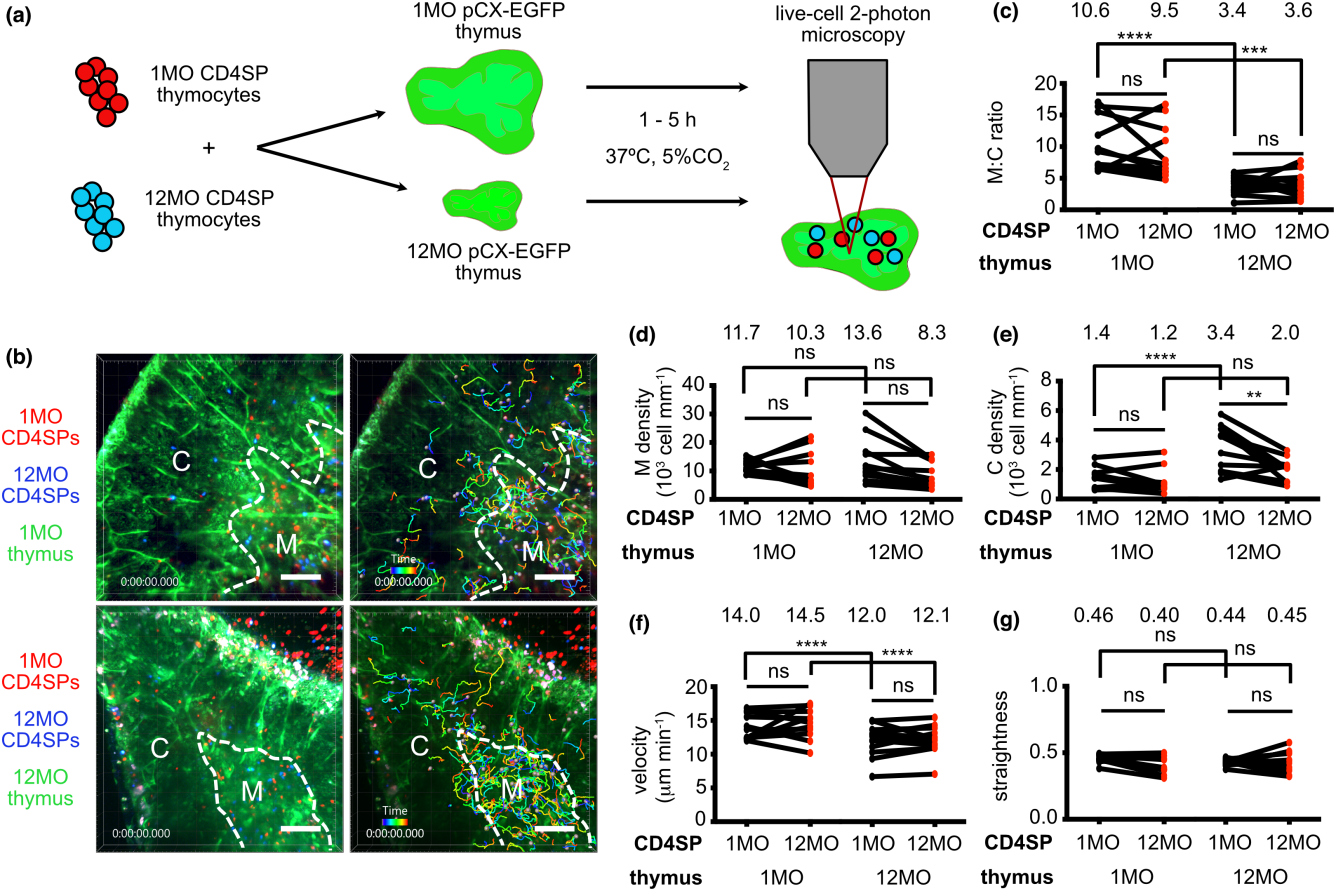
The middle‐aged thymus environment does not support rapid motility of medullary thymocytes. (a) Schematic of 2PM approach for imaging migration of CMPTX (red)‐ or Indo1AM (blue)‐labeled 1MO vs 12MO polyclonal CD4SP thymocytes in 1MO or 12MO live pCX‐EGFP (green) thymic slices. Time‐lapse imaging through a 40‐μm depth was carried out for 15 min to visualize CD4SP localization and migration. (b) Representative maximum intensity projections of 2PM imaging volumes at 20X magnification. The cortex (C) and medulla (M) are delineated by dashed white lines. 1MO (red) and 12MO (blue) CD4SP cells can be seen in the left images, while the right images show the corresponding cell tracks color‐encoded for elapsed imaging time. Scale bars, 100 μm. (c‐e) Quantification of the density of 1MO and 12MO CD4SP cells in the (c) medullary versus cortical imaging volumes (M:C ratio), (d) the medulla and (e) the cortex of 1MO versus 12MO thymic slices. (f) Mean cell velocity and (g) track straightness of 1MO and 12MO thymocytes migrating in 1MO versus 12MO thymic slices. Data are compiled from 4 experiments, with each symbol indicating the mean thymocyte value within a given thymic slice (*n*_1MO_ = 11, *n*_12MO_ = 12). Total cells tracked: *n*_1MO_ = 903 and *n*_12MO_ = 607 in 1MO slices, *n*_1MO_ = 1027 and *n*_12MO_ = 586 in 12MO slices. Analyzed by *t*‐tests, *p*‐values: ** <0.01, *** <0.001, **** <0.0001, ns: not significant. See also Figure S1 and Movies S1 and S2

To test whether the middle‐aged thymic environment supports rapid motility and efficient medullary accumulation of post‐positive selection thymocytes, we used 2PM to image young 1MO and middle‐aged 12MO polyclonal mouse CD4^+^ single‐positive (CD4SP) thymocytes migrating in 1MO or 12MO live thymic slices (Figure 1a). We first found that expression of CCR4 and CCR7, which promote medullary entry (Ehrlich et al., 2009; Hu et al., 2015; Ueno et al., 2004), are comparable in thymocytes of both ages (Figure S1a). CD4SPs were labeled with red or blue fluorescent dyes, and thymic slices were generated from pCX‐EGFP mice, in which cortical and medullary regions can be distinguished by cellular morphology and EGFP intensity (Lancaster & Ehrlich, 2017). Thymocyte migration was imaged by time‐lapse 2PM, and cells were tracked to determine their density in the medulla and cortex, as well their velocity and path straightness (Figure 1b and Movies [Link], [Link]).

The ratio of medullary to cortical densities of CD4SPs declined in the 12MO thymic environment, irrespective of thymocyte age (Figure 1c). However, the medullary density of CD4SP cells did not decline in 12MO thymuses (Figure 1d); instead, their density increased in the 12MO cortex, likely reflecting age‐associated cortical thinning (Chinn et al., 2012; Venables et al., 2019) (Figure 1e). Consistent with robust accumulation of CD4SP in the 12MO medulla, expression of CCL21, the CCR7 ligand required for CD4SP accumulation in the medulla (Kozai et al., 2017), was elevated at 12MO versus 1MO (Figure S1b). Notably, CD4SPs of both ages migrated significantly more slowly in the 12MO versus 1MO thymus (Figure 1f). Neither the age of thymocytes nor the thymic environment significantly impacted thymocyte straightness (Figure 1g). Thus, the middle‐aged thymus environment supports robust entry of CD4SP cells into the medulla but does not support their rapid migration, both of which enable efficient scanning of medullary APCs.

### 2.2 The middle‐aged thymic environment does not support efficient negative selection of CD8SP thymocytes responding to moderate‐avidity self‐antigens

**FIGURE 2:**
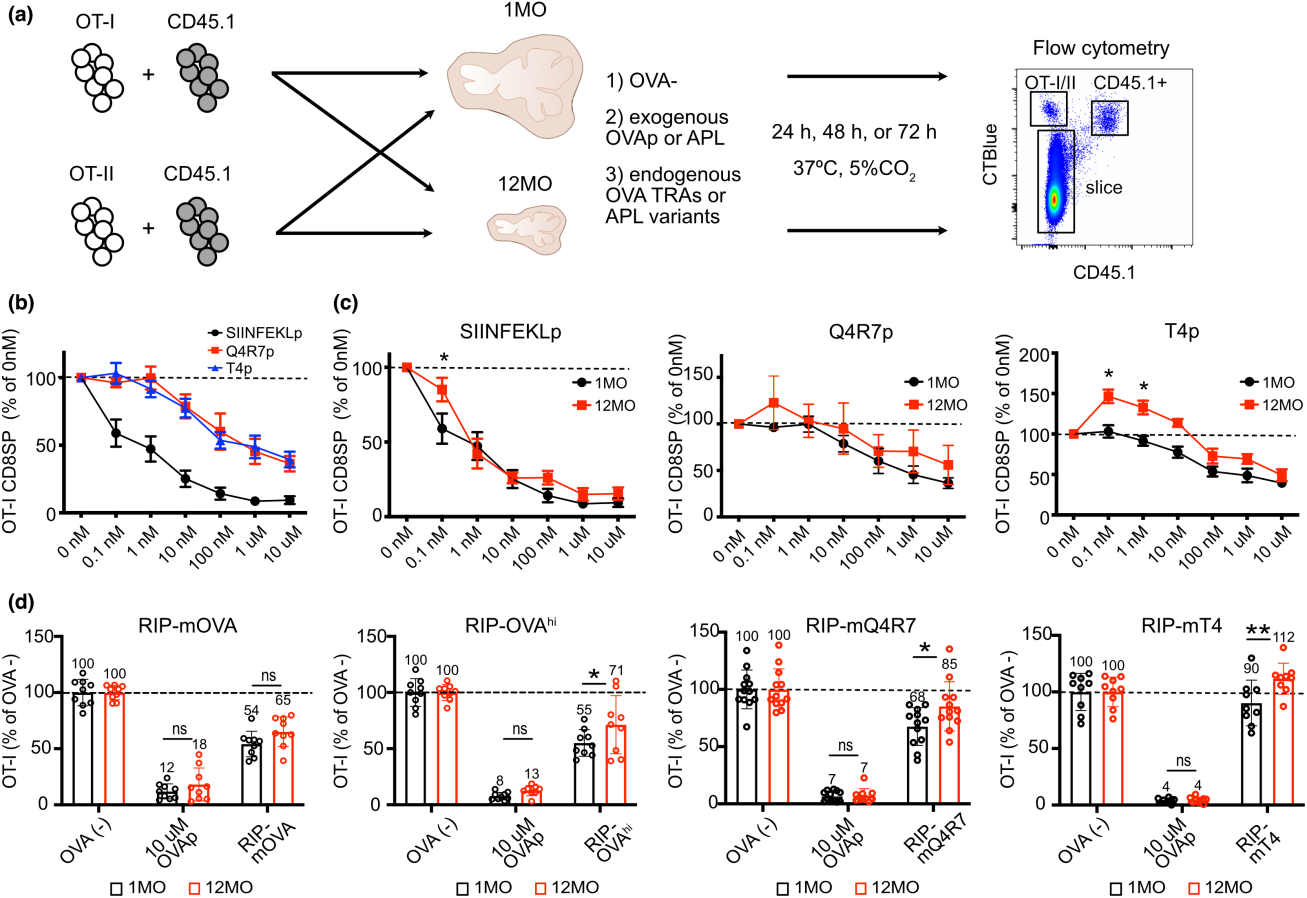
The 12MO thymic environment does not support efficient negative selection of OT‐I CD8SP thymocytes responding to moderate‐avidity self‐antigens. (a) Schematic of heterochronic slice deletion assays to assess negative selection of OT‐I or OT‐II thymocytes responding to ubiquitous self‐antigens or TRAs in young (1MO) versus middle‐aged (12MO) thymic slices. Slices were generated from (1) C57BL/6J wild‐type mice that did not express OVA (OVA^−^), (2) wild‐type mice followed by incubation with OVAp or lower affinity APLs, or (3) RIP‐mOVA, RIP‐OVA^hi^, RIP‐mQ4R7, or RIP‐mT4 model TRA transgenic mice. (b) The percent of OT‐I CD8SP cells remaining in 1MO thymic slices incubated overnight with the indicated concentrations of SIINFEKLp, Q4R7p, or T4p, relative to OT‐I CD8SP cells in slices incubated without peptide. Data are compiled from 6 experiments. (c) The percent of OT‐I CD8SP cells remaining in 1MO or 12MO thymic slices incubated with the indicated concentrations of SIINFEKLp, Q4R7p or T4p relative to those in slices without peptide. Data are compiled from 6–7 independent experiments. Data in (b) are a composite of the 1MO data shown in (c). (d) Negative selection of OT‐I CD8SP thymocytes responding to AIRE‐dependent TRAs in 1MO and 12MO thymic slices, evaluated after 48 h. The percent of OT‐I CD8SPs remaining in RIP‐mOVA, RIP‐OVA^hi^, RIP‐mQ4R7 and RIP‐mT4 thymic slices were quantified relative to OT‐I CD8SPs in OVA^‐^ thymic slices. Addition of 10 μM of SIINFEKLp (OVAp) served as a positive control for OT‐I negative selection. Data show mean ± SEM compiled from 3‐4 independent experiments per genotype, with three thymic slices per experiment. Each data point represents results from an individual thymic slice. Values were normalized to the mean of triplicate OVA^−^ slices in each experiment. Data in (c) and (d) were analyzed by two‐way ANOVA with Šídák's correction for multiple comparisons, *p*‐values: * <0.05, ** <0.01, ns: not significant

We next investigated whether central tolerance is impaired in the middle‐aged thymic environment using live thymic slice deletion assays. Negative selection of young 1MO CD8SP thymocytes responding to self‐antigens in 1MO versus 12MO thymic slices was quantified by flow cytometry (Figure 2a) (Hu et al., 2015; Lancaster et al., 2019). CD8SP negative selection was assayed with OT‐I TCR transgenic thymocytes, which express a TCR that recognizes a peptide of ovalbumin (OVAp), OVAp_257‐264_ (SIINFEKLp), presented by H‐2K^b^ (Hogquist et al., 1994). One advantage of the OT‐I system is that altered peptide ligands (APLs) of varying affinities for the OT‐I TCR have been defined and expressed as model TRAs under control of the rat insulin promoter (RIP) (Daniels et al., 2006; Koehli et al., 2014). To assay for negative selection, thymic slices (1) lacked ovalbumin (OVA‐), serving as negative controls for selection, (2) were incubated with OVAp or APLs, modeling ubiquitous self‐antigens, or (3) were generated from mice expressing OVA or APLs in mTECs under control of the *Aire*‐dependent RIP, modeling endogenous TRAs (Figure 2a). This assay allowed us to test the impact of an aged, non‐irradiated thymic environment on central tolerance without confounding differences between thymocyte ages. Also, by varying concentrations and/or TCR‐binding affinities of OVA peptides, the impact of TCR‐binding avidity could be directly assayed.

To test whether the middle‐aged thymus supports negative selection of CD8SP cells responding to ubiquitous self‐antigens, 1MO OT‐I thymocytes were introduced onto 1MO versus 12MO thymic slices incubated with varying concentrations of SIINFEKLp or OVA APLs. SIINFEKLp has a higher affinity for the OT‐I TCR (K_d_ 3.7 ± 0.7nM) than Q4R7p (K_d_ 48 ± 9.5 nM) or T4p (K_d_ 55 ± 10.1 nM), (Daniels et al., 2006). All three peptides induced negative selection in a concentration‐dependent manner after 24 hr on 1MO and 12MO thymic slices, and the efficiency of selection correlated with the peptide's TCR‐binding affinity (Figure 2b). Notably, at higher peptide concentrations, there was negligible difference in the extent of negative selection in 1MO versus 12MO thymic environments (Figure 2c). However, deletion on 12MO slices was significantly reduced at lower concentrations of SIINFEKLp and T4p, with a similar trend for Q4R7p (Figure 2c). Notably, in 12MO slices, the number of CD8SPs responding to 0.1–10 nM T4p exceeded that of the no‐peptide control, likely indicating a switch to positive selection in the presence of low concentrations of a weak agonist in the middle‐aged thymus environment. Together, these results indicate that the middle‐aged thymus becomes impaired in its ability to support negative selection of CD8SP thymocytes responding to low avidity ubiquitous self‐antigens.

To determine if the middle‐aged thymic environment supports negative selection of CD8SPs to endogenous TRAs, thymic slices were generated from RIP‐mOVA or RIP‐OVA^hi^ mice, expressing membrane‐bound or soluble forms of OVA, respectively (Kurts et al., 1996, 1998). These model TRAs induce OT‐I CD8SP negative selection *in vivo* (Gallegos & Bevan, 2004; Hubert et al., 2011) and in thymic slices (Lancaster et al., 2019). OVA^−^ littermate slices incubated without or with 10 μM OVAp served as negative and positive controls for deletion, respectively. Relative to 1MO thymic slices, the 12MO environment supported comparable OT‐I deletion to RIP‐mOVA. However, negative selection in 12MO slices was significantly impaired to the RIP‐OVA^hi^ TRA (Figure 2d). The RIP‐OVA^hi^ transgene is expressed at lower levels than RIP‐mOVA (Lancaster et al., 2019). In addition, the 12MO thymic environment did not support efficient negative selection of OT‐I cells responding to the lower affinity TRA variants RIP‐mQ4R7 or RIP‐mT4 (Figure 2d). Together, these data indicate that negative selection is impaired in the middle‐aged thymus for CD8SPs responding to TRAs of lower TCR‐binding avidities, due to either lower expression levels or reduced TCR‐binding affinities.

### 2.3 The middle‐aged thymic environment does not support efficient central tolerance of CD4SP thymocytes responding to lower‐avidity self‐antigens

**FIGURE 3:**
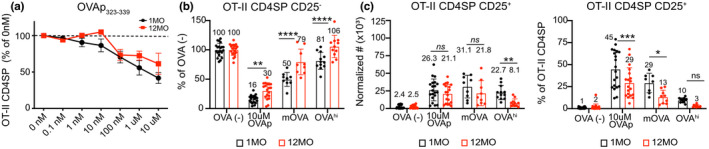
The 12MO thymic environment does not support efficient negative selection of OT‐II CD4SP thymocytes responding to ubiquitous self‐antigens or TRAs. (a) The percent of OT‐II CD4SP cellularity following incubation overnight in 1MO or 12MO thymic slices with the indicated concentrations of OVAp_323‐339_ relative to OT‐II CD4SP cells remaining in slices incubated without OVAp. Plots show mean ± SEM of compiled data from four independent experiments. Analyzed by two‐way ANOVA with Šídák's correction for multiple comparisons. (b) Negative selection of OT‐II CD4SP thymocytes and (c) induction of CD25^+^ Treg‐P responding to the indicated TRAs on 1MO or 12MO thymic slices were quantified. (b) The percent of OT‐II CD4SP cells remaining in RIP‐mOVA and RIP‐OVA^hi^ thymic slices relative to OVA‐ slices. Addition of 10 μM of OVAp_323‐339_ (OVAp) served as a positive control for OT‐II negative selection. (c) Normalized cell numbers (left) and frequencies (right) of CD25+ OT‐II CD4SP cells. Data in (b‐c) show mean ± SEM of thymic slices, compiled from 3‐4 independent experiments, with three thymic slices per experiment. Each data point represents an individual thymic slice. Analyzed by two‐way ANOVA with Šídák's correction for multiple comparisons, *p*‐values: * <0.05, ** <0.01, *** <0.001, **** <0.0001, ns: not significant. See also Figure S2

To determine if the middle‐aged thymus supports efficient negative selection of CD4SP thymocytes, 1MO OT‐II thymocytes, which recognize OVAp_323‐339_ presented by I‐A^b^ (Barnden et al., 1998), were introduced into 1MO versus 12MO thymic slices with varying concentrations of OVAp_323–339_. Slices of both ages induced concentration‐dependent negative selection of OT‐II CD4SPs after 24hr (Figure 3a). Notably, 12MO thymic slices were impaired in their ability to support negative selection of OT‐II thymocytes to 10 μM OVAp as well as the TRAs RIP‐mOVA and RIP‐OVA^hi^ after 48 h (Figure 3b). Thus, negative selection of CD4SP cells responsive to both ubiquitous self‐antigens and TRAs is impaired in the 12MO thymic environment.

We also assessed whether diversion of OT‐II CD4SP thymocytes toward the Treg lineage was impaired in the 12MO thymic environment. While the number of OT‐II CD4SP CD25^+^ Treg precursors (Treg‐P) (Hsieh et al., 2004) generated in a middle‐aged thymus did not decline significantly in response to 10 μM OVAp or the RIP‐mOVA TRA after 48 hr, the frequency of CD4SP cells upregulating CD25 was significantly diminished (Figure 3c and Figure S2a). These findings indicate that CD4SP cells can be induced, albeit less efficiently, to divert toward a Treg fate when strong self‐antigens are presented ubiquitously or as TRAs in the middle‐aged thymus. However, in response to the less abundant RIP‐OVA^hi^ TRA, the number of OT‐II CD4SP CD25^+^ Treg‐P declined significantly in the 12MO thymic environment (Figure 3c and Figure S2a). Thus, diversion of autoreactive OT‐II CD4SPs toward the Treg fate is inefficient in response to lower‐avidity TRAs in the middle‐aged thymic environment.

### 2.4 The middle‐aged thymus does not support efficient Treg induction to TRAs

**FIGURE 4:**
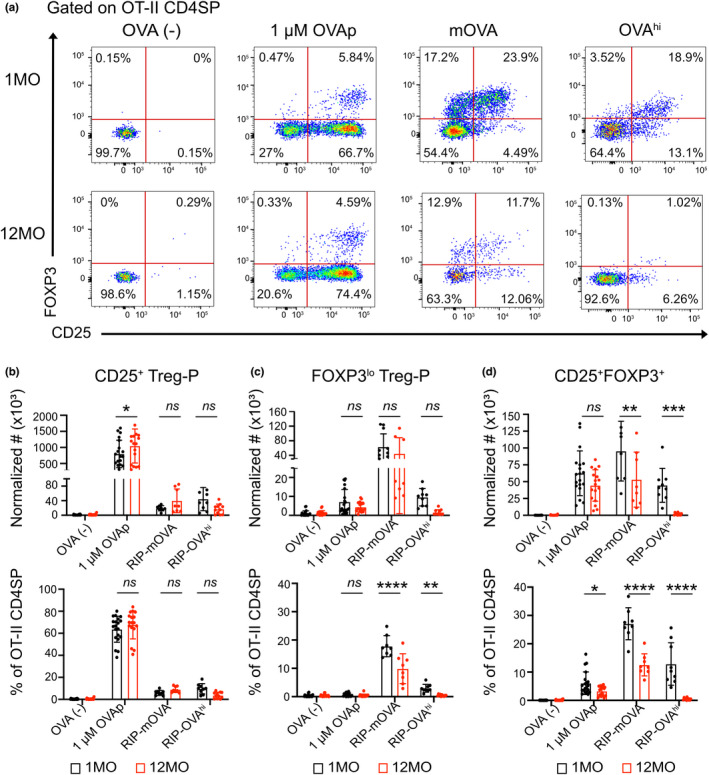
The middle‐aged thymic environment does not support efficient induction of Tregs in response to TRAs. (a) Representative flow cytometry plots of Treg precursors (CD25^+^ Treg‐P and FOXP3^lo^ Treg‐P) and Tregs (CD25^+^FOXP3^+^) recovered from OVA(‐), WT slices with addition of 1 μM of OVAp_323‐339,_ RIP‐mOVA, and RIP‐OVA^hi^ 1MO versus 12MO thymic slices after 72 hr. Normalized cell numbers (top) and percentages (bottom) of (b) CD25^+^ Treg‐P, (c) FOXP3^lo^ Treg‐P and (d) Treg CD4SP OT‐II subsets were quantified. Data in (b‐d) show mean ± SEM compiled from 3‐4 independent experiments, with three thymic slices per experiment. Each data point represents one thymic slice. Values were normalized to the mean of triplicate OVA‐ slices. Analyzed by two‐way ANOVA with Šídák's correction for multiple comparisons, *p*‐values: * <0.05, ** <0.01, *** <0.001, **** <0.0001, ns: not significant

Previous studies showed that by 12MO of age, the mouse thymus generates few Treg (Thiault et al., 2015). Because the 12MO thymic environment supported induction of OT‐II CD25^+^ CD4SP Treg‐P in response to ubiquitous self‐antigens, but not the RIP‐OVA^hi^ TRA (Figure 3c), we wondered if the age‐associated decline in *de novo* Treg differentiation preferentially impacts thymocytes responding to a subset of self‐antigens. Thus, Treg differentiation was assayed in 1MO versus 12MO thymic slices after 72 hr to enable sufficient time for FOXP3 upregulation (Weist et al., 2015). We also assayed differentiation of the two Treg‐P populations, CD25^+^FOXP3^−^ (CD25^+^) Treg‐P and CD4^+^CD25^−^FOXP3^lo^ (FOXP3^lo^) Treg‐P, as these two subsets differ in their developmental programs and capacity to prevent autoimmunity. Relative to Foxp3^lo^ Treg‐P, CD25^+^ Treg‐P become mature Treg with faster kinetics after positive selection, have higher affinities for self‐antigens, and undergo increased rates of apoptosis. Foxp3^lo^ Treg‐P are uniquely dependent on *Nfkb1* and require IL‐4 signaling for efficient maturation. In addition, the TCR repertoires differ for these two progenitors, and Treg derived from CD25+ Treg‐P, but not Foxp3^lo^ Treg‐P, protect against EAE (Owen et al., 2019). To assay for Treg differentiation on thymic slices, we used OT‐II thymocytes that contained virtually no detectable Tregs or Treg‐P (Figure S2b). In the absence of cognate antigen (OVA^−^), OT‐II thymocytes did not differentiate into Treg‐P or Tregs (Figure 4a). In the presence of 1μM OVAp_323‐339_, middle‐aged 12MO slices supported generation of CD25^+^ Treg‐P and Treg as efficiently as young slices, although there was a decline in Treg frequency (Figure 4a,b,d). Notably, ~60% of OT‐II CD4SP thymocytes upregulated CD25 in response to OVAp, regardless of the age of the thymic microenvironment (Figure 4a,b), indicating that access to ubiquitous antigens is not impaired in the middle‐aged thymus. Together, these results demonstrate that the middle‐aged thymic environment supports fairly efficient *de novo* generation of CD25^+^ Treg‐P and Treg to ubiquitously presented self‐antigens.

Next, we tested the efficiency of Treg‐P differentiation in response to the *Aire*‐dependent RIP‐mOVA and RIP‐OVA^hi^ TRAs. 12MO slices supported CD25^+^ Treg‐P differentiation to TRAs as well as 1MO slices, although fewer CD25^+^ Treg‐P were generated in response to TRAs compared to OVAp on slices of both ages (Figure 4a,b). Interestingly, the RIP‐mOVA TRA induced the highest frequency and number of FOXP3^lo^ Treg‐P (Figure 4c). Notably, the frequency of FOXP3^lo^ Treg‐P declined significantly in the middle‐aged thymic environment in response to both RIP‐mOVA and RIP‐OVA^hi^ TRAs (Figure 4a,c).

Differentiation of CD25+ FOXP3+ Tregs in response to TRAs was the most significantly impaired in the naturally aged thymic environment. The RIP‐mOVA TRA induced efficient Treg generation in 1MO slices, in which ~25% of remaining OT‐II CD4SP thymocytes at 72 h were Tregs. Both the number and frequency of Tregs were markedly lower in middle‐aged RIP‐mOVA slices (Figure 4d). An even starker decline in Treg induction was observed in middle‐aged slices expressing the less abundant RIP‐OVA^hi^ TRA, in which mature Tregs were almost undetectable (Figure 4d). Together, these results demonstrate that the middle‐aged thymic environment is impaired in its capacity to support Treg generation to TRAs, but maintains the ability to support Treg differentiation to ubiquitous self‐antigens.

### 2.5 Aging associated changes in central tolerance of polyclonal thymocytes

**FIGURE 5:**
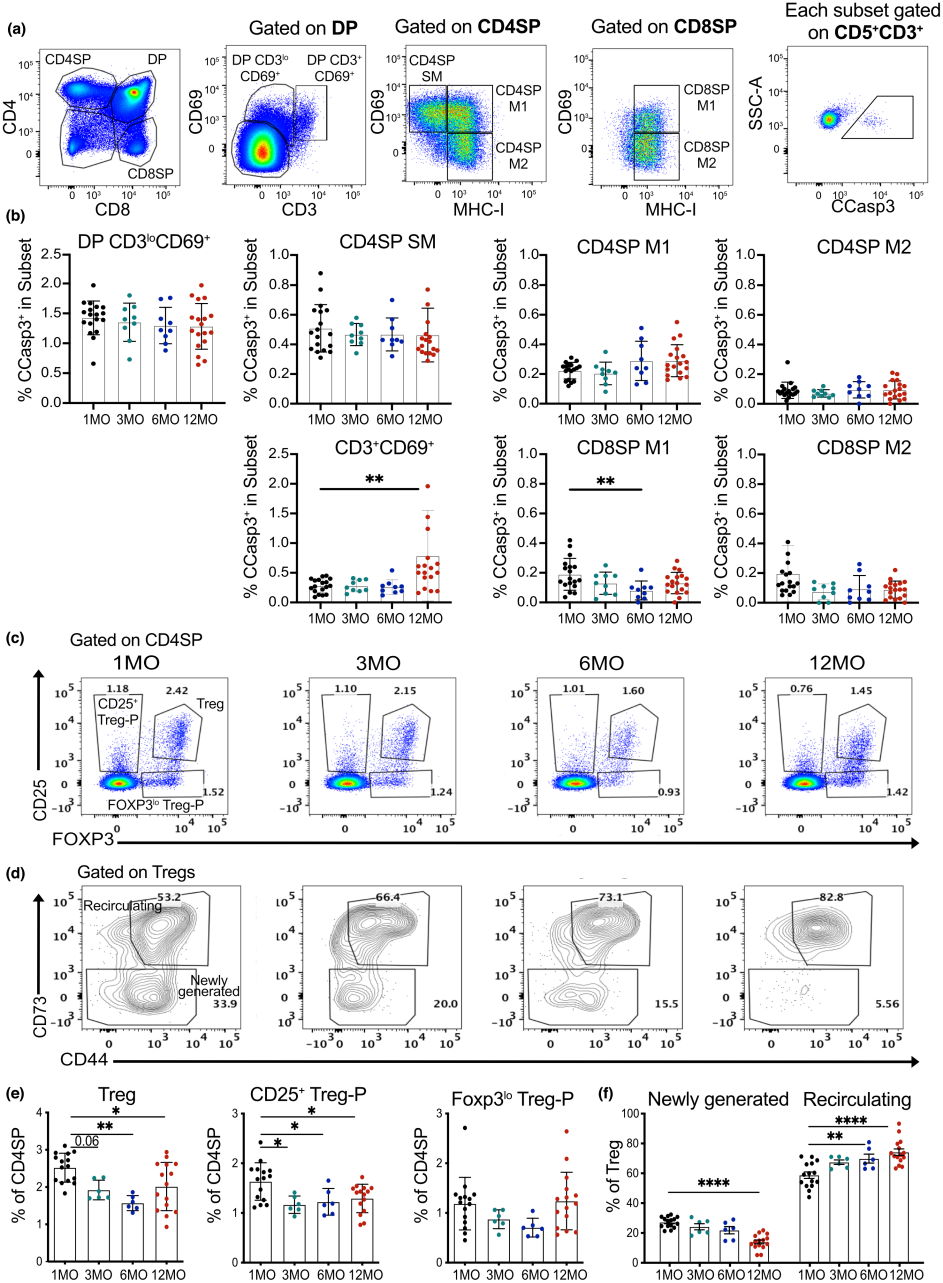
Aging associated changes in central tolerance of polyclonal thymocytes. (a) Representative flow cytometry plots showing identification of thymocyte subsets undergoing negative selection. Post‐positive selection DP thymocytes were subdivided into DP CD3^lo^CD69^+^ and DP CD3^+^CD69^+^ stages; CD4SP and CD8SP cells were divided into semi‐mature (SM), mature1 (M1) and mature2 (M2) stages, as indicated. Cells were gated on CD5^+^ CD3^+^ cells to restrict analysis to thymocytes that had undergone TCR signaling, and cleaved caspase 3 (CCasp3) expression identified cells undergoing clonal deletion in each subset. (b) Quantification of the frequency of CCasp3+ cells in thymocyte subsets from mice at 1, 3, 6 and 12 MO of age. (c‐d) Representative flow cytometry plots of (c) CD25 and FOXP3 to distinguish Treg‐P and Tregs and (d) CD73 on Tregs to distinguish newly generated (CD73^−^) from recirculating (CD73^+^) cells in thymuses from mice at 1, 3, 6 and 12 MO of age. (e) Percentage of Tregs, CD25^+^ Treg‐Ps, and FOXP3^lo^ Treg‐Ps in the CD4SP compartment at the indicated ages. (f) Percentage of newly generated versus recirculating Tregs at the indicated ages. (b, e‐f) Plots show mean ± SEM of nine to fifteen thymuses per age. Symbols represent individual thymuses. Analyzed by one‐way ANOVA with Tukey's test for multiple comparisons, *p*‐values: * <0.05, ** <0.01, *** <0.001, **** <0.0001

To determine if the age‐associated decline in central tolerance observed with antigen‐specific models is evident in polyclonal thymocytes, we quantified negative selection and Treg induction in mice from 1MO to 12MO of age. We assessed the frequency of post‐positive selection thymocyte subsets undergoing apoptosis, as identified by intracellular cleaved caspase 3 (CCasp3). CD4^+^CD8^+^ double positive (DP) thymocytes were subdivided into early post‐positive selection CD3^lo^CD69^+^ cells and later CD3^+^CD69^+^ cells. CD4SP and CD8SP subsets were divided into semi‐mature (SM), mature 1 (M1), and mature (M2) cells based on expression of CD69 and MHC‐I (Xing et al., 2016) (Figure 5a). Analysis was restricted to cells that had received a TCR signal (CD5^+^CD3^+^), and the frequency of Ccasp3^+^ cells was quantified. The less mature DP CD3^lo^CD69^+^cells and CD4SP SM subsets underwent higher rates of negative selection than more mature SP subsets. Although there was a decline in the frequency of CD8SP M1 cells undergoing negative selection at 6MO of age, we did not observe a decline in the frequency of any polyclonal subsets undergoing negative selection by 12MO of age (Figure 5b). Thus, overall rates of negative selection are relatively constant in the thymus from 1MO through 12MO of age, likely reflecting ongoing negative selection to abundant ubiquitous self‐antigens.

We also tested if there was a decline in the generation of polyclonal Tregs and Treg‐P by 12MO of age (Figure 5c). Previous studies indicated that *de novo* Treg induction in an aging thymus is impaired due to an increased number of peripheral Tregs that recirculate into the thymus where they outcompete newly differentiating Tregs for limited, local IL‐2, which is required for Foxp3 upregulation (Hemmers et al., 2019; Thiault et al., 2015). To quantify *de novo* polyclonal Treg generation with age, we distinguished newly generated from recirculating Tregs based on CD73 expression (Owen et al., 2019) (Figure 5d). The frequency of Tregs within the CD4SP compartment diminishes significantly by 6MO of age (Figure 5e). Within the thymic Treg compartment, the frequency of newly generated cells steadily declines over 12MO of age, with a concomitant increase in recirculating Tregs (Figure 5d,f), consistent with previous studies (Thiault et al., 2015). These findings indicate that the age‐associated decrease in generation of Tregs can be detected in the polyclonal repertoire, consistent with our observation that Treg induction was severely compromised for OT‐II thymocytes responding to TRAs and was somewhat impaired for cells responding to a ubiquitous a self‐antigen in a 12MO thymic environment (Figure 4).

The proportion of CD25^+^ Treg‐P within the CD4SP compartment decreases significantly with age, with a trend toward diminished frequencies of Foxp3^lo^ Treg‐P as well (Figure 5e). Because CD25 upregulation is induced by TCR activation (Lio & Hsieh, 2008), the age‐associated reduction in CD25^+^ Treg‐P is consistent with reduced thymocyte access to self‐antigens that promote Treg differentiation. The concept that self‐antigen availability limits the induction of Tregs in a middle‐aged thymus is concordant with the finding that OT‐II thymocytes generate Treg‐P fairly efficiently in a 12MO thymic environment in response to abundant ubiquitous self‐antigens, but not to lower abundance endogenous TRAs (Figure 4).

### 2.6 The composition of TECs and HAPCs is altered in a middle‐aged thymus

**FIGURE 6:**
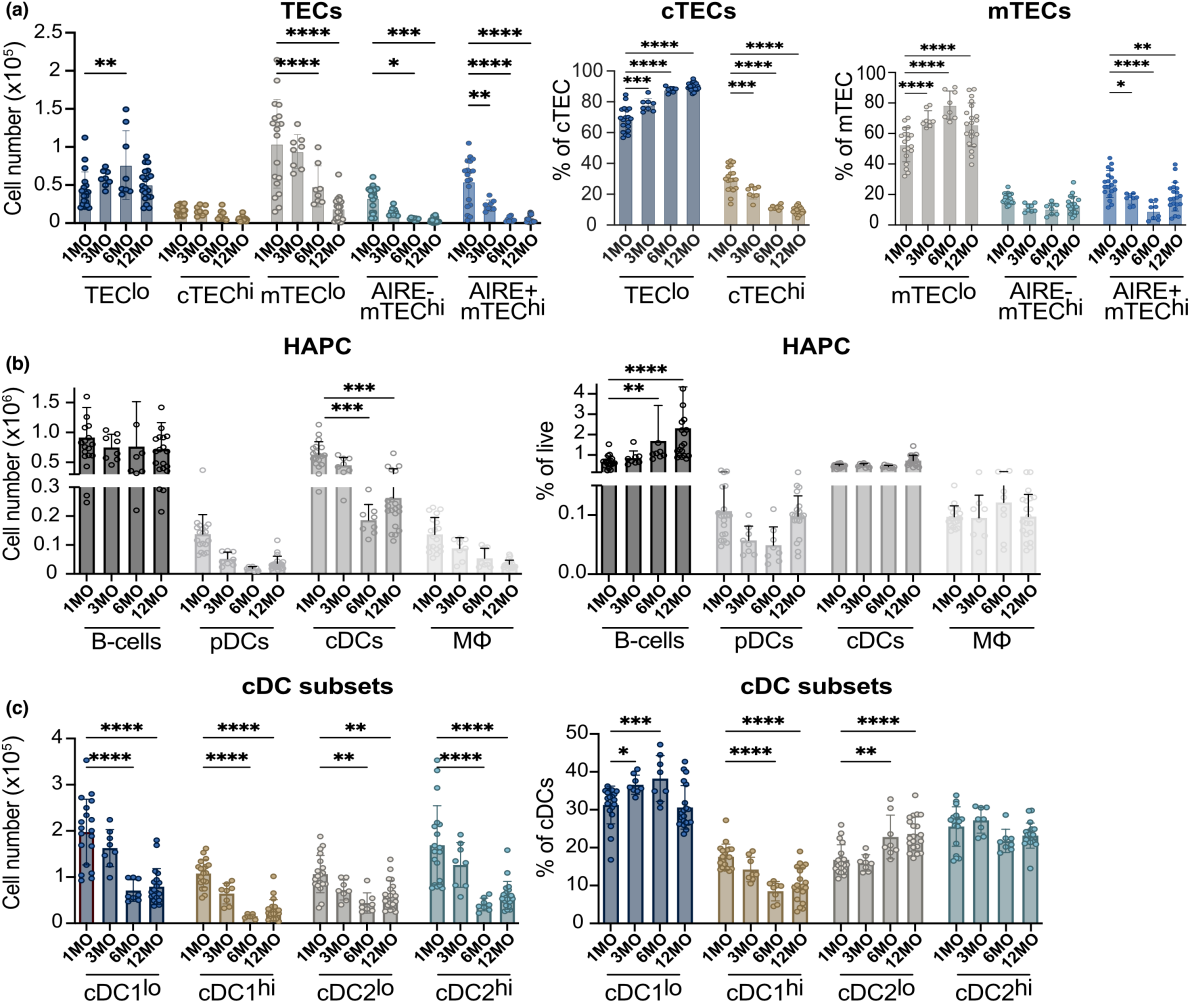
The composition of TEC and HAPC compartments is significantly altered in a middle‐aged thymus. (a) Total cellularity and frequency of TEC subsets in 1, 3, 6 and 12MO thymuses were quantified by flow cytometry. (b) The total number and percentage HAPC subsets were quantified in 1, 3, 6 and 12MO thymuses. TECs, B cells, pDC, cDCs and macrophages (mΦ) were gated as in Figure S3. (c) The total number and percentage of thymic cDC subsets were quantified at 1, 3, 6 and 12MO of age. Plots show mean ± SEM of 8–20 thymuses per age. Data are compiled from 6 experiments. Analyzed by *t*‐test, *p*‐values: * <0.05, ** <0.01, *** <0.001

Given the reduced capacity of the middle‐aged thymus to support efficient negative selection and Treg induction to self‐antigens of moderate avidities, we tested whether aging alters the cellular composition of TECs and HAPCs, the major APC subsets critical for promoting central tolerance to diverse self‐antigens. Thymic TECs, B cells, pDCs, cDCs, and macrophages were quantified by flow cytometry in mice at 1, 3, 6, and 12MO of age. cDCs were divided into cDC1 and cDC2 subsets, and TECs were divided into cTECs and mTECs. Both TEC and cDC subsets were further subdivided based on low versus high MHC‐II expression, and AIRE^+^ cells were identified within CD80^+^MHCII^hi^ mTECs (Figure S3). The number of TEC^lo^ cells increased from 1MO to 6MO, with a slight reduction in cTEC^hi^ cells by middle age (Figure 6a). Increased TEC^lo^ numbers were reflected in their increased frequency in the cTEC population, with a commensurate decrease in cTEC^hi^ frequencies by middle age (Figure 6a). All mTEC subsets declined numerically at 6 and 12MO of age, consistent with the overall decline in thymus cellularity, but only the AIRE^+^ mTEC^hi^ subset was reduced in frequency within the mTEC compartment (Figure 6a). Thus, by middle‐age, thymic involution is associated with a shift in TEC composition toward TEC^lo^ and mTEC^lo^ subsets, with a substantial decrease in the frequency of AIRE^+^ mTECs, the subset that expresses diverse TRAs.

The HAPC compartment also undergoes significant changes by middle‐age. The number of B cells did not decline by 12MO, resulting in an increased frequency of total thymocytes (Figure 6b), consistent with previous studies (Cepeda et al., 2018). The number of cDCs decreased significantly by 6 and 12 MO of age (Figure 6b), consistent with the overall decline in thymus cellularity, as their frequency remained constant with age (Figure 6b). Similarly, pDCs and macrophages decreased somewhat in number but not in frequency by middle‐age (Figure 6b). Overall, broad HAPC subsets decreased at approximately the same rate as total thymus cellularity through middle‐age, with the exception of thymic B cells, which increased in frequency.

As distinct cDC subsets have been differentially implicated in tolerance induction (Ardouin et al., 2016; Leventhal et al., 2016; Oh et al., 2017; Perry et al., 2014, 2018), age‐associated changes in cDC subsets were further evaluated. Within XCR1^+^ cDC1s, the number of cDC1^lo^ and cDC1^hi^ subsets decreased significantly by 12MO of age. Interestingly, the frequency of cDC1^hi^ cells in the cDC compartment decreased, while cDC1^lo^ cells increased with age (Figure 6c). cDC1^hi^ cells have been implicated in acquiring self‐antigens from *Aire* ^+^ mTECs to induce Treg selection (Perry et al., 2014). Both cDC2^lo^ and cDC2^hi^ numbers also decreased significantly with age (Figure 6c). However, cDC2^lo^ constituted an increasing proportion of total cDCs with age (Figure 6c). cDC2s have also been implicated in presenting *Aire*‐dependent antigens to induce Treg selection (Leventhal et al., 2016). Thus, similar to changes in the TEC compartment, the cDC compartment becomes enriched for MHCII^lo^ cDC1 and cDC2 subsets by middle age, consistent with reduced presentation of diverse self‐antigens that induce thymocyte tolerance.

## 3 DISCUSSION

Here we identify specific defects in central tolerance that manifest in the middle‐aged thymus. Following peak thymus size and T‐cell output at 1MO of age in mice, thymic cellularity and T‐cell export progressively decline (Chinn et al., 2012; Srinivasan et al., 2021). During age‐associated thymus involution, TECs become less proliferative, their numbers diminish, and their cellular composition and organization change. The number of AIRE^+^ mTECs and expression of TRAs decline by 12MO of age, (Baran‐Gale et al., 2020; Bredenkamp et al., 2014; Gray et al., 2006; Griffith et al., 2012; Lepletier et al., 2019; Venables et al., 2019), suggesting central tolerance to TRAs could be particularly impaired by middle‐age, but this possibility had not been evaluated. In this study, we used naturally aged thymic slices to directly test if the middle‐aged thymic environment retains the capacity to support central tolerance to ubiquitous self‐antigens and/or TRAs. We determine that if thymocytes encounter high‐avidity self‐antigens, negative selection is largely preserved in a middle‐aged thymus. However, the 12MO thymic environment does not induce efficient negative selection or Treg differentiation for thymocytes encountering lower‐avidity self‐antigens, regardless of whether they are presented ubiquitously throughout the thymus or in the medulla as TRAs. Thus, by middle‐age, the thymus is selectively impaired in its capacity to induce central tolerance to moderate‐avidity self‐antigens.

Thymocytes must efficiently enter the medulla to scan APCs that present the diverse self‐antigens, including *Aire*‐dependent TRAs, that establish broad self‐tolerance to autoantigens throughout the body. After positive selection, thymocytes upregulate the chemokine receptors CCR7 and CCR4, which promote their accumulation in the medulla where the corresponding chemokine ligands are expressed (Ehrlich et al., 2009; Hu et al., 2015; Ueno et al., 2004). Thus, we considered that as TEC organization deteriorates with age, thymocyte medullary accumulation could be compromised. However, the middle‐aged thymic environment supported robust accumulation of CD4SPs in the medulla. Interestingly, CCL21, the CCR7 ligand responsible for attracting thymocytes into the medulla (Kozai et al., 2017), is expressed at higher levels in the medulla of middle‐aged relative to young thymuses. Thus, our data indicate that impaired central tolerance in the middle‐aged thymus is not due to reduced thymocyte medullary accumulation.

Given that thymocyte residence is limited to about 5 days in the medulla (McCaughtry et al., 2007), where diverse self‐antigens are displayed in a sparse mosaic (Baran‐Gale et al., 2020; Derbinski et al., 2005, 2008; Meredith et al., 2015; Sansom et al., 2014), medullary thymocytes need to migrate rapidly to encounter sufficient APCs to ensure complete tolerance. We find that the velocity of CD4SP cells is significantly reduced in a middle‐aged 12MO versus young 1MO thymic environment, regardless of thymocyte age. This reduced speed could limit the number of APCs thymocytes encounter in a middle‐aged thymus, diminishing the efficiency of central tolerance. Future studies are needed to determine why thymocyte migration is slower in a 12MO thymus.

Because TRA expression declines with age, and our data and previous studies show that the number and frequency of AIRE^+^ mTECs are reduced (Baran‐Gale et al., 2020; Bredenkamp et al., 2014; Griffith et al., 2012; Lepletier et al., 2019), we hypothesized that an age‐associated deficiency in negative selection would be most appreciable for TRAs. At first glance, our data seem consistent with this conclusion in that the middle‐aged thymic environment largely supported negative selection of OT‐I CD8SP cells to exogenously administered ubiquitous SIINFEKLp, while the efficiency of negative selection to the RIP‐OVA^hi^ TRA was significantly reduced by about 30%. However, ubiquitous versus *Aire*‐dependent tissue‐restricted expression of a self‐antigen was not sufficient to predict whether tolerance induction would be impaired in a middle‐aged thymic environment. For example, negative selection of OT‐I CD8SP thymocytes to the RIP‐mOVA TRA was intact in 12MO thymic slices, while negative selection of OT‐II CD4SP thymocytes to ubiquitous OVAp was impaired.

A better predictor of whether negative selection was impaired in a middle‐aged thymic environment was the avidity of the selecting self‐antigen for the TCR, which is determined by the amount of self‐antigen encountered by thymocytes and its TCR‐binding affinity. We evaluated negative selection using altered peptide ligands for the OT‐I TCR that are reported to be just below (T4), just above (Q4R7), or well above (SIINFEKL) the TCR affinity threshold for clonal deletion (Daniels et al., 2006). OT‐I negative selection in the middle‐aged thymus was intact in response to high concentrations of all three self‐antigens but was impaired in the presence of low concentrations of both high and low affinity peptides. Moreover, only the lowest concentration of high‐affinity SIINFEKLp revealed a deficit in negative selection in the 12MO thymus, while this deficiency was apparent with higher concentrations of the lower affinity T4p. These findings show that the middle‐aged thymic environment becomes less efficient at displaying ubiquitous self‐antigens to induce negative selection, effectively raising the avidity threshold for negative selection by middle‐age. The mechanisms underlying the deficit remain to be determined, but could reflect changes in APC composition, such as the reduced frequency of MHCII^hi^ cDCs.

The middle‐aged thymus was also deficient in inducing negative selection against lower‐avidity TRAs. Negative selection of OT‐I thymocytes in 12MO thymic environments was intact to the higher affinity RIP‐mOVA TRA, but was significantly impaired to the lower affinity variants RIP‐mQ4R7 and RIP‐mT4, as well as to the high affinity, but lower abundance RIP‐OVA^hi^ TRA (Kurts et al., 1998; Lancaster et al., 2019). Taken together, these findings further support that negative selection to lower‐avidity self‐antigens, even when expressed as TRAs, is impaired in the middle‐aged thymus. Mechanisms underlying diminished negative selection to TRAs have yet to be determined, but could reflect a combination of slower thymocyte migration in the medulla, as discussed above, reduced numbers and frequencies of AIRE^+^ mTECs that express and present TRAs (Aschenbrenner et al., 2007; Hinterberger et al., 2010; Lancaster et al., 2019), and reduced MHCII^hi^ cDC1s that have been implicated in acquiring antigens from AIRE^+^ mTECs for display to thymocytes (Perry et al., 2014, 2018).

Although the decline in negative selection observed in the middle‐aged thymus may seem modest, even a small number of self‐reactive T cells in the periphery can induce autoimmunity (Bosch et al., 2017). Interestingly, when the low affinity T4p was expressed as a TRA or presented as a less abundant ubiquitous self‐antigen in 12MO thymic slices, we consistently recovered more OT‐I CD8SPs than in control slices without antigen. T4p has been shown to induce negative selection at high concentrations and positive selection at lower doses (Daniels et al., 2006). Thus, by middle age, the thymus may induce positive selection of thymocytes specific for low avidity self‐antigens, perhaps resulting in export of an elevated number of autoreactive T cells to the periphery. Importantly, OT‐I CD8 T cells that escape negative selection to the low avidity TRAs RIP‐mQ4R7 and RIP‐mT4 potently induce autoimmune diabetes upon immunization with OVAp or infection with OVA‐expressing *Listeria monocytogenes* (Koehli et al., 2014). Future experiments will investigate if OT‐I CD8SP that escape negative selection in the middle‐aged thymus prime heightened autoimmune responses at this age. Furthermore, although we did not find general evidence of impaired negative selection of polyclonal thymocytes at 12MO of age, the frequency of CD8SP M1 cells undergoing clonal deletion did decline with age. Because negative selection in the middle‐aged thymus is impaired selectively to moderate‐avidity self‐antigens, intact negative selection to high‐avidity self‐antigens could mask the deficit in polyclonal cells. Altogether, these findings suggest impaired negative selection in a middle‐aged thymus could result in export of weakly autoreactive T cells that could induce autoimmunity, perhaps in the context of pathogen mimicry, contributing to the peak in new‐onset autoimmunity in middle age (Watad et al., 2017). Future studies will test this possibility.

The middle‐aged thymic environment was particularly impaired in supporting generation of new Tregs responsive to TRAs; however, Tregs differentiated efficiently in response to a ubiquitous self‐antigen. The 12MO thymic environment supported efficient Treg generation against ubiquitous OVAp, which was surprising as previous studies indicated new Treg development is limited by IL‐2 availability due to competition from recirculating peripheral Tregs (Thiault et al., 2015; Weist et al., 2015). Tregs in the thymus can arise from two distinct progenitors, CD25^+^ Treg‐P or FOXP3^lo^ Treg‐P (Owen et al., 2019). In a two‐step process, thymocytes can first receive a TCR signal that upregulates the high‐affinity IL‐2 receptor α‐chain (CD25), generating CD25^+^ Treg‐P. Subsequent IL‐2 signaling induces *Foxp3* expression and Treg differentiation (Burchill et al., 2008; Lio & Hsieh, 2008). Alternatively, thymic Tregs can arise via FoxP3^lo^ Treg‐P, which differ from CD25^+^ Treg‐P in their transcriptome, TCR repertoire, developmental kinetics, susceptibility to apoptosis, dependence on cytokines, and suppressive functions (Owen et al., 2019; Tai et al., 2013). Exogenous OVAp induced robust CD25 expression by OT‐II CD4SPs in 1MO and 12MO thymi. If IL‐2 were limiting in the 12MO thymus, fewer CD25^+^ Treg‐P would have been expected to upregulate FOXP3 and differentiate into Treg. However, the 12MO environment generated a comparable number of Treg as the 1MO environment, despite the observed increase in recirculating Treg. In contrast, there was a significant reduction in the frequency of OT‐II CD4SP that differentiated into FOXP3^lo^ Treg‐P in the 12MO thymic environment in response to TRAs. Notably, the frequency and number of OT‐II Tregs declined significantly in response to both RIP‐mOVA and RIP‐OVA^hi^ TRAs in the 12MO thymus; in fact, Treg induction to RIP‐OVA^hi^ was virtually extinguished. Altogether, these findings suggest a revised model in which access to TRAs is a key limiting factor for Treg induction in the aging thymus environment. Access to cytokines that can contribute to Treg differentiation, like IL‐4 (Owen et al., 2019), or local concentrations of IL‐2 could also be limiting. The reduced frequencies of AIRE^+^ mTECs and MHCII^hi^ cDC1s in the middle‐aged thymic environment, both of which express and/or present *Aire*‐dependent TRAs to autoreactive thymocytes (Ardouin et al., 2016; Aschenbrenner et al., 2007; Gallegos & Bevan, 2004; Hinterberger et al., 2010; Hubert et al., 2011; Lancaster et al., 2019; Perry et al., 2014, 2018), suggest that an altered APC compartment is a major contributor to reduced selection of new Tregs in the aging thymus. It remains to be resolved how age‐associated changes in APC subsets impact negative selection and Treg induction to distinct self‐antigens in the young versus middle‐aged thymus.

## 4 EXPERIMENTAL PROCEDURES

### 4.1 Mice

C57BL/6J (Jackson Laboratories and NIH/NIA), B6.SJL‐Ptprc^a^Pepc^b^/BoyJ (CD45.1), C57BL/6‐Tg(TcraTcrb)1100Mjb/J (OT‐I)(Hogquist et al., 1994), B6. Cg‐Tg(TcraTcrb)425Cbn/J (OT‐II)(Barnden et al., 1998), C57BL/6‐Tg(Ins2‐TFRC/OVA)296Wehi/WehiJ (RIP‐mOVA)(Kurts et al., 1996), RIP‐OVA^hi^ (W. R. Heath, University of Melbourne, Melbourne, Australia)(Kurts et al., 1998), RIP‐mT4 (E. Palmer, University of Basel, Basel, Switzerland), RIP‐mQ4R7 (E. Palmer, University of Basel, Basel, Switzerland) and pCX‐EGFP (I. Weissman, Stanford University, Stanford, CA)(Wright et al., 2001) strains were bred in‐house. All strains were sourced from Jackson Laboratories, except as specified. Mouse maintenance and experimental procedures were carried out with approval from the Institutional Animal Care and Use Committee at UT Austin. All strains were bred and maintained under specific pathogen‐free conditions in the UT Austin animal facility.

### 4.2 Thymic slice preparation

For 2PM imaging, 400 μm live thymic slices were vibratome‐sectioned from pCX‐EGFP thymi, and for negative selection assays, slices were generated from C57BL/6, RIP‐mOVA, RIP‐OVA^hi^, RIP‐mT4, or RIP‐mQ4R7 thymi (Lancaster & Ehrlich, 2017). Slices were collected in DRPMI +10% bovine calf serum on ice before transfer to 0.4‐μm tissue culture inserts (Millipore) in 35‐mm Petri dishes containing 1 mL of complete RPMI medium, with or without added peptides.

### 4.3 Two‐photon fluorescence microscopy

CD4SP cells were enriched from 1MO and 12MO thymi and stained with CMTPX CellTracker Red or 2 μM Indo1AM dyes (Life Technologies), prior to incubation for ≥1 h on pCX‐EGFP thymic slices. Slices were imaged (Lancaster et al., 2019) every 15 s, through a depth of 40 μm, at 5‐μm intervals for durations of 15 min, using an Ultima IV microscope (Bruker) with a 20× water immersion objective (NA 1.0) and PrairieView software (v.5.4, Bruker). The sample was illuminated with two MaiTai titanium:sapphire lasers (Newport). Migratory cell paths were tracked, and mean cell velocities and path straightness calculated using Imaris (v9, Bitplane). Cell densities were quantified in manually demarcated cortical and medullary regions at the first time point for each dataset.

### 4.4 Selection assays in thymic slices

10^6^ OT‐I or OT‐II thymocytes and 10^6^ CD45.1 thymocytes per slice, along with the input control, were stained with 5 μM CMF2HC CellTracker Blue (Life Technologies) and applied to thymic slices generated from C57BL/6J, RIP‐mOVA, RIP‐OVA^hi^, RIP‐mT4, or RIP‐mQ4R7 mice, in the presence or absence of the indicated concentrations of OVAp (OVA_257‐264_ for OT‐I, New England Peptide; or OVA_323‐339_ for OT‐II, GenScript), T4p (Anaspec) or Q4R7p (GenScript) for specified durations. Cells were quantified by flow cytometry and normalized for variable slice entry based on the ratio of control polyclonal CD45.1^+^ cells in each slice to the comparable CD45.1^+^ cells in the input sample. Triplicate slices of each condition were analyzed per experiment. Data were normalized to the average number of cells in OVA‐ slices in the same experiment to quantify negative selection or Treg induction.

See Supplementary Experimental Procedures in Appendix S1 for more information.

## CONFLICT OF INTEREST

None declared.

## AUTHOR CONTRIBUTIONS

J.L., D.K.‐C., and L.E. designed the experiments and wrote the manuscript; J.L., D.K.‐C., J.S., and Y.L. performed experiments and analyzed data; H.S. and S.N. performed experiments; E.R. and L.E. edited the manuscript.

## Supporting information

Appendix S1

Movie S1

Movie S2

## Data Availability

Data are available upon request from the corresponding author.
