# Supplementary material for: Central tolerance is impaired in the middle‐aged thymic environment
Source: Aging Cell. 2022 May 13;21(6):e13624. doi: 10.1111/acel.13624 (PMC9197411; doi:10.1111/acel.13624)
Supplement: Supplementary file 1 — Appendix S1 [file ACEL-21-e13624-s003.pdf]

SUPPLEMENTARY INFORMATION

Central tolerance is impaired in the middle-aged thymic microenvironment

J. N. Lancaster, D. L. Keatinge-Clay, J. Srinivasan, Y. Li, H. J. Selden, S. Nam, E. R. Richie, and L. I. R. Ehrlich

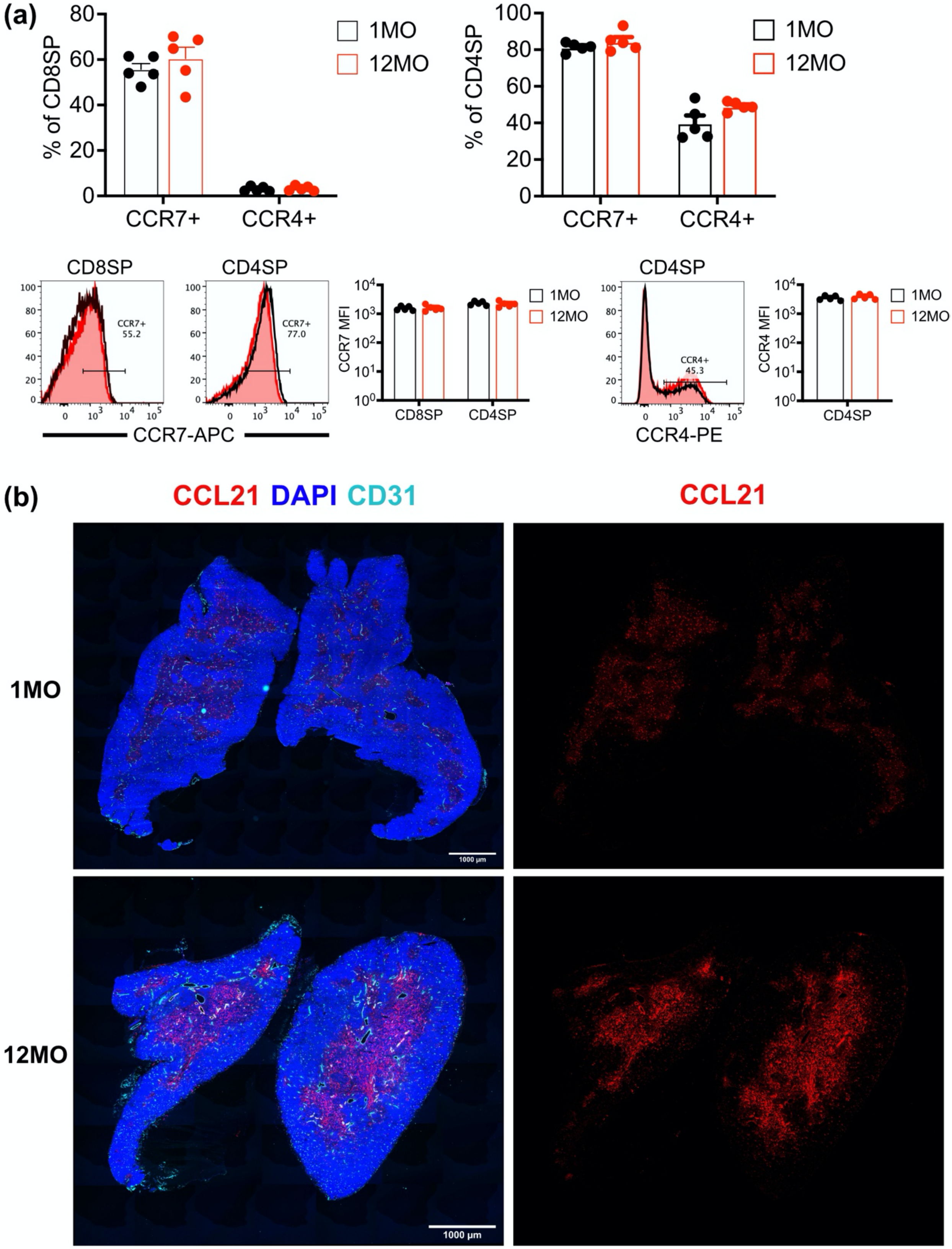
(a)
% of CD8SP
CCR7+
CCR4+
1MO
12MO
% of CD4SP
CD8SP
CD4SP
CCR7+
55.2
CCR7+
77.0
CCR7-APC
CCR7 MFI
CCR4+
45.3
CCR4-PE
CCR4 MFI
(b)
CCL21 DAPI CD31
CCL21
1MO
12MO
1000 μm
1000 μm

Supplementary Figure 1, related to Figure 1. Expression of CCR4, CCR7, and CCL21 do not decline in 12MO versus 1MO thymuses. (a) The proportion of CD8SP and CD4SP thymocytes that express the chemokine receptors CCR7 and CCR4 in 1MO (black) and 12MO (red) thymuses were evaluated by flow cytometry (top row). Histograms of CCR7 and CCR4 expression on CD8SP and/or CD4SP populations are shown, with gates delineating receptor-expressing cells; MFIs for receptor-positive cells were quantified(bottom row). The threshold for CCR7 expression was set based on lack of expression in $CD4^{-}CD8^{-}$ thymocytes. Each data point represents a mouse, with bars showing the means + SEM. **(b)** Immunofluorescence images of 1MO and 12MO thymus sections, immunostained for CCL21 (red) and CD31 (cyan), with a DAPI nuclear counterstain (blue). Staining and display parameters were kept constant to reveal differences in expression levels of CCL21 in 1 vs 12 mo thymi. Scale bar is 1000 μm.

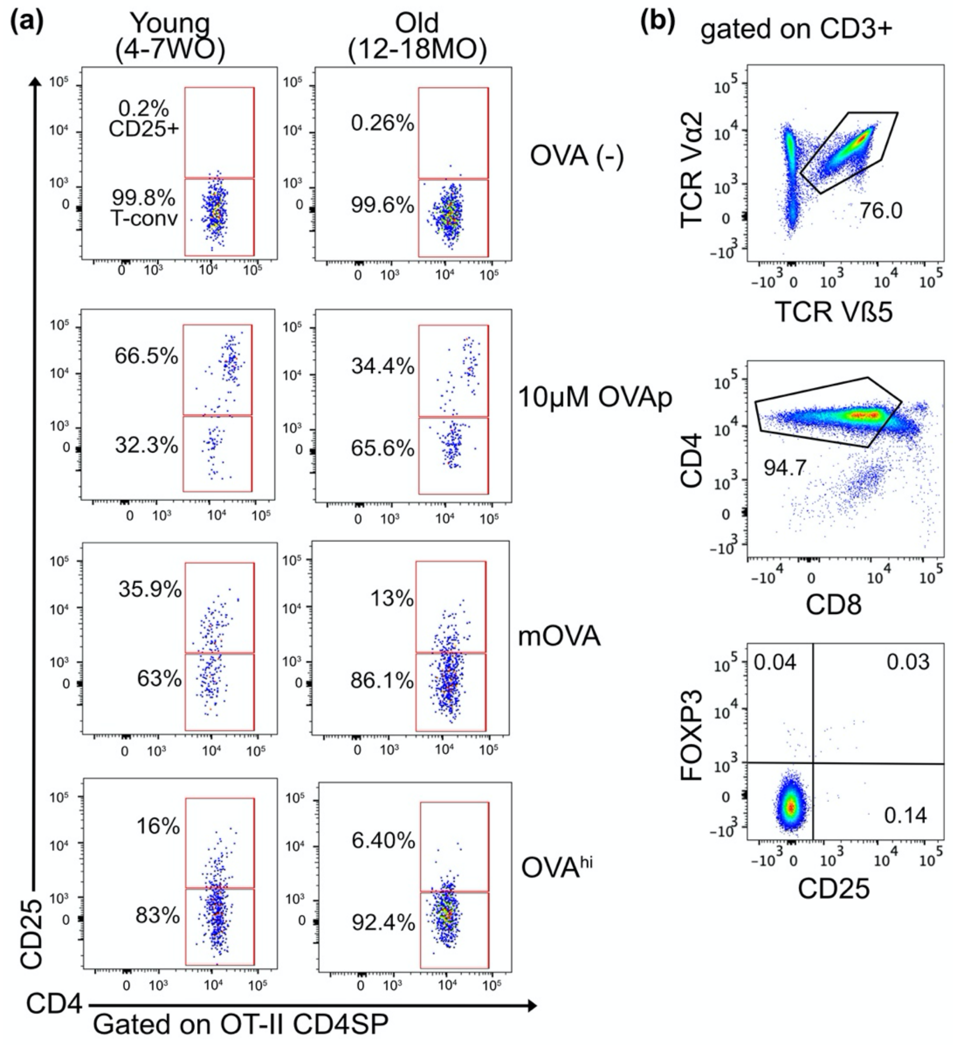

Supplementary Figure 2, related to Figure 3. Deletion and Treg induction of OT-II CD4SPs against exogenous and endogenous self-antigens. (a) Representative flow cytometry plots gated to show OT-II CD4SP CD25$^-$ and OT-II CD4SP CD25+ cells recovered from OVA(-), RIP-mOVA (mOVA) and RIP-OVA^{hi} (OVA^{hi}) live thymic tissue slices from young vs. aged mice, at 48 h post-incubation. 10 μM of OVAp$_{323-339}$ (OVAp) was added as a positive control for OT-II negative selection. **(b)** Sequential gating scheme for flow cytometric analysis of OT-II thymocytes shows lack of Treg and Treg-P in input OT-II thymocytes used in Treg generation assays.

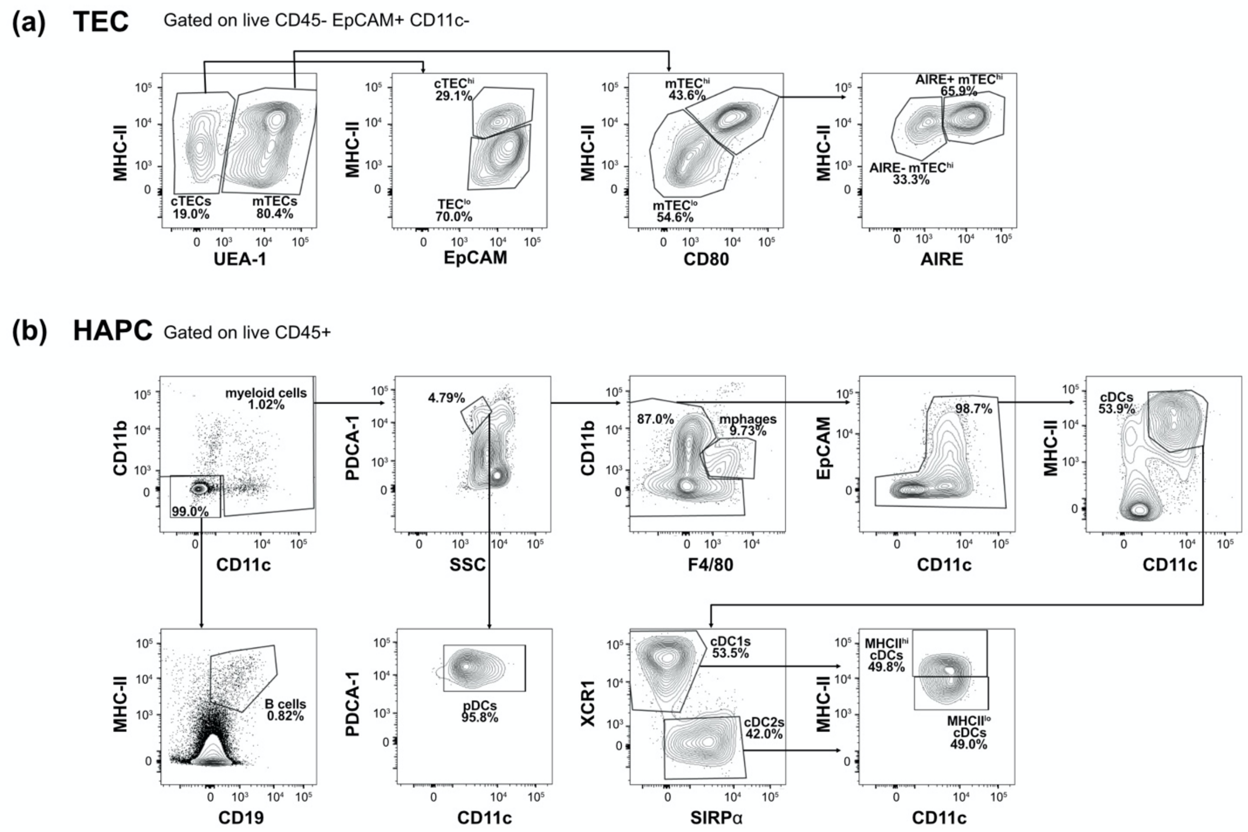

Supplementary Figure 3, related to Figure 6. Flow cytometric analyses of TECs and HAPCs. (a) Representative flow cytometry plots to quantify the frequency of cTEC and mTEC subsets within enzymatically digested 1MO and 12MO thymuses: TEC^{lo} (CD45^{-}EpCAM^{+}CD11c^{-}UEA-1^{-}MHC-II^{lo}), cTEC^{hi} (CD45^{-}EpCAM^{+}CD11c^{-}UEA-1^{-}MHC-II^{hi}), mTEC^{lo} (CD45^{-}EpCAM^{+}CD11c^{-}UEA-1^{+}MHC-II^{lo}), AIRE^{-} mTEC^{hi} (CD45^{-}EpCAM^{+}CD11c^{-}UEA-1^{+}MHC-II^{hi}AIRE^{-}), AIRE^{+} mTEC^{hi} (CD45^{-}EpCAM^{+}CD11c^{-}UEA-1^{+}MHC-II^{hi}AIRE^{+}). **(b)** Representative flow cytometry plots to quantify the frequency of HAPC subsets within enzymatically digested 1MO and 12MO thymuses: B cell (CD45^{+}CD11b^{-}CD11c^{-}CD19^{+}MHC-II^{+}), pDC (CD45^{+} PDCA-1^{+}CD11c^{int}SSC^{lo}), macrophages (CD45^{+}CD11b^{+}F4/80^{+}), cDC1 (CD45^{+}F4/80^{-}CD11c^{+}MHC-II^{+}XCR1^{+}), cDC2 (CD45^{+} F4/80^{-}CD11c^{+}MHC-II^{+}SIRPα^{+}). cDC1 and cDC2 subsets were further subdivided into MHC-II^{lo} and MHC-II^{hi} populations.

SUPPLEMENTARY MOVIE LEGENDS

Supplementary Movie 1, related to Figure 1. Migration of 1MO and 12MO CD4SP thymocytes on a 1MO thymus slice. 1MO (red) and 12MO (blue) CD4SPs migrate within an EGFP-expressing 1MO thymus slice (left panel). Thymocyte track times are color-encoded as indicated (right panel). Images were acquired for 15 min with 15 sec time intervals, through a depth of 40 μm, and a maximum intensity projection is displayed. Scale bar is 100 μm.

Supplementary Movie 2, related to Figure 1. Migration of 1MO and 12MO CD4SP thymocytes on a 12MO thymus slice. 1MO (red) and 12MO (blue) CD4SPs migrate within an EGFP-expressing 12MO thymus slice (left panel). Thymocyte track times are color-encoded as indicated (right panel). Images were acquired for 15 min with 15 sec time intervals, through a depth of 40 μm, and a maximum intensity projection is displayed. Scale bar is 100 μm.

Supplementary Table 1: Antibodies for flow cytometry and immunofluorescence

Cell Marker	Clone	Fluorophore	Source	Catalog Number
CD3	17A2	PECy7	BioLegend	100220
CD4	RM4-5	BV510	BioLegend	100559
CD8	53-6.7	FITC	BioLegend	100706
CD11b (Mac-1)	M1/70	AlexaFluor 700	BioLegend	101222
CD11b (Mac-1)	M1/70	Purified	BioXCell	BE0007
CD11c	N418	Pacific Blue	BioLegend	117322
CD16/32	93	Purified	BioLegend	101301
CD19	1D3/CD19	PerCPCy5.5	BioLegend	152405
CD25	PC61	APC	BioLegend	102012
CD31	MEC13.3	Biotinylated	BioLegend	102503
CD44	IM7	AlexaFluor 700	BioLegend	103026
CD45	30-F11	BV510	BioLegend	103138
CD45.1	A20	APC	BioLegend	110714
CD69	H1.2F3	Biotinylated	BioLegend	104504
CD73	TY/11.8	PECy7	BioLegend	127223
CD80	16-10A1	Pacific Blue	eBioscience	48-0801-80

AIRE	5H12	FITC	eBioscience	53-5934-82
B220 (CD45R)	RA3.3A1/6.1	Purified	BioXCell	BE0067
CCL21	Goat polyclonal	Purified	R&D Systems	AF457
CCR4	2G12	PE	BioLegend	131204
CCR7	4B12	APC	BioLegend	120108
Cleaved Caspase 3	D3E9	AlexaFluor 488	Cell Signaling	9603
EpCAM	G8.8	PE	BioLegend	118206
F4/80	BM8	APCCy7	BioLegend	123118
FOXP3	FJK-16S	PE	Invitrogen	12-5773-80
Goat IgG	Polyclonal	AlexaFluor 647	Jackson Immunoresearch	705-605-147
GR-1 (Ly6G)	RB6-8C5	Purified	BioXCell	BE0075
I-A/I-E (MHC-II)	M5/114.15.2	PECy7	BioLegend	107630
Keratin 5	Poly19055	Purified	BioLegend	905501
MHC-I (H-2K^b)	AF6-88.5	PerCPCy5.5	BioLegend	116516

PDCA-1	927	AlexaFluor 488	BioLegend	127012
Rabbit IgG	Polyclonal	AlexaFluor 594	Jackson Immunoresearch	711-586-152
SIRPα (CD172)	P84	APC	BioLegend	144014
TCR Vα2	B20.1	PECy7	BioLegend	127822
TCR Vβ5	MR9-4	FITC	BioLegend	139514
TER-119	TER-119	Purified	BioXCell	BE0183
XCR1	ZET	Biotinylated	BioLegend	148212

Supplementary Experimental Procedures

Thymic slice preparation

For 2PM imaging, slices were generated from pCX-EGFP thymi. For negative selection assays, slices were generated from C57BL/6, RIP-mOVA, RIP-OVAhi, RIP-mT4, or RIP-mQ4R7 thymi. Dissected thymi were embedded in 4% (w/v) NuSieve GTG low-melting-temperature agarose (Lonza) in PBS at 37°C. The solidified agarose block was sectioned into 400-μm-thick slices using a VT 1000 S Microtome (Leica) in a bath of ice-cold PBS, with vibratome frequency set to 70 Hz, speed to 0.20 mm s^{-1}, and amplitude to 0.6 mm. Slices were collected in DRPMI + 10% bovine calf serum on ice before transfer to 0.4-μm tissue culture inserts (Millipore) in 35-mm Petri dishes containing 1 mL of complete RPMI medium, with or without added peptides.

Two-photon fluorescence microscopy

After incubation for ≥ 1 h, pCX-EGFP thymic slices were transferred and secured in an imaging chamber (Harvard Apparatus) on the microscope stage. Perfusion medium, consisting of DRPMI supplemented with 2 g L^{-1} sodium bicarbonate, 5 mM HEPES, and 1.25 mM calcium chloride, was gravity fed to the stage inlet through a 300-mL IV set, and circulated through the imaging chamber at a flow rate at ~100 mL h^{-1}, or ~1 drop per second. The perfusion medium was aerated with 95% oxygen and 5% carbon dioxide and maintained at 37°C with a heated microscope stage and inline perfusion heater. Images were acquired every 15 s, through a depth of 40 μm, at 5-μm intervals for durations of 15 min, using an Ultima IV microscope (Bruker) with a 20× water immersion objective (NA 1.0) and PrairieView software (v.5.4, Bruker). The sample was illuminated with two MaiTai titanium:sapphire lasers (Newport) tuned to 750 nm (for Indo1AM) and

900 nm (for CMTPX and EGFP). Emitted light was passed through 473/24, 525/50, and 605/70 band-pass filters (Chroma) to separate GaAsP detectors for detection of Indo1 (blue), EGFP, and CMTPX (red) fluorescence, respectively.

Migratory paths for thymocytes were tracked, and mean cell velocity and path straightness calculated using Imaris (v9, Bitplane). The enrichment of thymocytes in the medulla was determined at the first time point for each dataset by measuring the number of thymocytes in manually demarcated cortical and medullary regions.

Negative selection assays in thymic slices

10^6 OT-I or OT-II thymocytes and 10^6 CD45.1 thymocytes per slice, along with the input control, were stained in 5 mL of DRPMI medium supplemented with 0.2 g L^{-1} sodium bicarbonate and 20 mM HEPES, with 5 μM CMF2HC CellTracker Blue (Life Technologies). Cells were washed and resuspended in 5 mL of complete RPMI medium for 30 min to destain, and then washed twice before application onto thymic slices generated from C57BL/6J, RIP-mOVA, RIP-OVA^{hi}, RIP-mT4, or RIP-mQ4R7 mice. Slices were incubated in 37°C/5% CO_2 on tissue culture inserts in Petri dishes containing 1 mL of complete RPMI, with or without OVAp ($OVA_{257-264}$ for OT-I, New England Peptide; or $OVA_{323-339}$ for OT-II, GenScript), T4p (Anaspec) or Q4R7p (GenScript) peptides for specified durations.

For analysis, slices were gently washed twice by submerging in PBS and manually disrupted to obtain single-cell suspensions. Input thymocytes and slice samples were stained with the following fluorophore-conjugated antibodies: anti-CD3, -CD4, -CD8, -CD25, -TCR Vα2, -TCR Vβ5, and -CD45.1 (Table 1). Samples were washed and resuspended in 10 μg mL^{-1} PI for viability. For Treg induction experiments, samples were

stained with fluorophore-conjugated antibodies against surface markers and Zombie Red viability dye (BioLegend) as described above, and fixed and permeabilized using the FOXP3/Transcription Factor Fix Perm kit (Tonbo Biosciences) per manufacturer instructions. Intracellular FOXP3 was stained by fluorophore-conjugated antibody for 20 min on ice, washed, and resuspended in PBS. After staining, 5×10^4 polystyrene beads were added to the tubes for cell quantification and flow cytometric analysis was carried out. Cell subsets were quantified and normalized for variable slice entry based on the ratio of control CD45.1$^+$ cells in each slice to the comparable CD45.1$^+$ cells in the input sample. Triplicate slices of each condition were analyzed in each experiment. Data were normalized to the average number of cells in OVA- slices in the same experiment.

Antibodies

Antibodies used in flow cytometry and immunofluorescence analyses are detailed in **Supplementary Table 1**. For flow cytometry, 5-10 $\times 10^6$ cells were immunostained in 100 μL of PBS + 2% bovine calf serum (BCS) with fluorochrome-conjugated antibodies. Unless otherwise specified, cells were incubated with fluorochrome-conjugated antibodies for 20 min on ice, washed twice in PBS + 2% BCS, incubated with streptavidin for 20' on ice, washed and resuspended in 10 μg mL^{-1} propidium iodide (PI) to determine viability.

Purification of thymocytes for live-cell microscopy

CD4SP cells were enriched by incubating 2×10^8 cells mL^{-1} with antibodies against CD8 (96 μg mL^{-1}) and CD11b, GR-1, TER-119, and CD25 (5 μg mL^{-1} each) for 30 min on ice in PBS + 2% BCS, followed by immunomagnetic depletion using sheep anti-rat IgG magnetic DynaBeads (Life Technologies at a 2:1 cell:bead ratio. Magnetic depletion was

repeated with half the number of beads to improve enrichments. Purity of isolated CD4SP was determined by flow cytometry using the following fluorochrome-conjugated antibodies: anti-CD3, -CD4, -CD8, and -CD69, followed by Streptavidin Qdot605 (Thermo Fisher Scientific, 1:800 dilution). Cells were washed and resuspended in 10 μg mL^{-1} propidium iodide (PI) to determine viability. Samples were analyzed on an LSR Fortessa flow cytometer (BD Biosciences), and data were analyzed using FlowJo (v.10, TreeStar).

For each slice, 10^6 isolated cells were stained with either 2 μM CMTPX CellTracker Red or 2 μM Indo1AM (both from Life Technologies) for 30 min at 37°C in 1.5 mL of DRPMI medium (RPMI 1640 without L-glutamine, phenol red, and sodium bicarbonate; Cellgro) supplemented with 0.2 g L^{-1} sodium bicarbonate and 20 mM HEPES. Cells were washed and incubated in 1.5 mL complete RPMI medium (RPMI 1640 with 2 mM L-glutamine, 50 U mL^{-1} penicillin, 50 mg mL^{-1} streptomycin, and 10% (v/v) fetal bovine serum) for 30 min to destain. Cells were washed, combined so that 10^6 CellTracker Red-labeled cells and 10^6 Indo1AM-labeled cells were mixed into each tube, and washed again with complete RPMI medium. Thymocytes were concentrated into 20-μL complete RPMI medium and carefully pipetted onto the surface of each thymic slice before incubation at 37°C 5% CO_2 to allow migration of thymocytes into the thymic slice. To avoid color channel bias, 1MO and 12MO thymocyte fluorophores were swapped in different experiments.

Flow-cytometric analyses of thymic stroma

Dissected thymi were cut into small fragments and enzymatically digested in 2-mL PBS with 2.5 mg/mL Liberase (Roche) with 120 μL DNase I solution [10,000 U, Roche, in 10 mL 50% glycerol : 50% DNase buffer (40 mM Tris-HCl +100 mM NaCl + 200 μg mL⁻

[1] bovine serum albumin)] for 12 min at 37°C, gently swirling halfway through incubation. The supernatant was transferred into 35 mL of PBS + 2% bovine calf serum and 5 mM EDTA at 4°C, with digestion of the remaining tissue fragments repeated twice to completely dissociate the tissue. The cells were spun down and filtered through a 70-μm nylon mesh to achieve a single-cell suspension.

For TEC analyses, cells were stained with 1 μg mL^{-1} biotinylated *Ulex europaeus* agglutinin I (UEA-1; Vector Laboratories) and the following fluorophore-conjugated antibodies: anti-CD11c, -CD45, -CD80, -EpCAM, and -I-A/I-E, and Zombie Red viability dye. Secondary staining was conducted with streptavidin conjugated Qdot 605 (1:400, Invitrogen) on ice for 20 min, followed by washing and fixation and permeabilization with the FOXP3/Transcription Factor Fix Perm kit. Intracellular AIRE was stained by fluorophore-conjugated antibody for 30 min on ice, washed, and resuspended in PBS. Quantification of TEC subsets was conducted by flow cytometry.

For HAPC analyses, cells were stained with the following fluorophore-conjugated antibodies: anti-CD11b, -CD11c, -CD19, -F4/80, -I-A/I-E, -PDCA-1, -SIRPα -XCR1. After washing, secondary staining was conducted by incubating with streptavidin-conjugated Qdot 605 (1:400, Invitrogen) on ice for 20 min. Samples were washed and resuspended in 10 μg mL^{-1} PI for viability. Quantification of HAPC subsets was conducted by flow cytometry.

Flow cytometric analyses of thymocytes

Single-cell suspensions of thymocytes were obtained by manually dissociating dissected thymi and filtering cells through 40-μm cell strainers. Samples were immunostained with the following fluorophore-conjugated antibodies: anti-CD3, -CD4, -

CD8, -CD69, -MHC-I, -CCR4, -CCR7 for 30 min at 37°C, prior to washing and resuspending the cells in 10 μg mL^{-1} PI for viability. To quantify negative selection within the thymus, thymocytes were immunostained with the following fluorophore-conjugated antibodies and dye: anti-CD3, -CD4, -CD5, -CD8, -CD69, -MHC-I and Zombie Red Viability dye (1:1000, BioLegend) for 30 min on ice. Cells were washed, then fixed and permeabilized using the Cytofix/Cytoperm kit (BD Biosciences BDB554714) per manufacturer instructions. Intracellular cleaved caspase 3 was stained with fluorophore-conjugated anti-cleaved caspase 3 antibody for 30 min on ice, washed, and resuspended in PBS for flow acquisition. Quantification of thymocyte subsets was conducted by flow cytometry.

To analyze polyclonal Tregs, single-cell thymocyte suspension were immunostained with the following fluorophore-conjugated antibodies: anti-CD3, -CD4, -CD8, -CD25, -CD44, -CD73 and Zombie Red viability dye (1:1000 dilution, BioLegend) at 4°C, and then fixed and permeabilized using the FOXP3/Transcription Factor Fix Perm kit (Tonbo Biosciences) per manufacturer instructions. Intracellular FOXP3 was immunostained with a fluorophore-conjugated antibody for 20 min on ice, and cells were washed and resuspended in PBS. Quantification of thymocyte subsets was conducted by flow cytometry.

Immunofluorescence analysis of thymic cryosections

Thymuses were embedded in Tissue-Tek OCT medium and frozen using a mixture of dry ice and isopentane. 7-μm cryosections were generated with CryoStar NX50 cryostat (ThermoFisher) and stored at -80ºC. Prior to immunostaining, sections were fixed in 100% acetone at -20ºC for 20 minutes, washed with PBS + 0.1% Tween-20, and

blocked with a solution of 10mM Tris HCl, 150mM NaCl and 0.5% blocking reagent (Perkin Elmer TSA Biotin System kit component). Fc receptor blocking was carried out with a 30-min room temperature stain using 10 μg mL^{-1} α-CD16/32. The sections were then incubated overnight at 4 ºC with 10 μg mL^{-1} α-CCL21, 5 μg mL^{-1} α-CD31-biotin, and 1.66 μg mL^{-1} α-Keratin 5. Following the overnight stain, the sections were incubated for 1 h at room temperature with fluorophore-conjugated secondary antibodies and streptavidin-conjugated Alexa Fluor 488 (Life Technologies, S11223). 4',6-diamidino-2-phenylindole (DAPI; Life technologies) was used at 0.125 μg mL^{-1} in PBS to stain nuclei.

Statistics

All statistical analyses were performed using Prism (GraphPad) with the corresponding test and multiple-test corrections listed in the Figure Legends.
